# Assessing kinship detection: single nucleotide polymorphism array density and estimator comparison in white-tailed deer

**DOI:** 10.1093/g3journal/jkag007

**Published:** 2026-01-25

**Authors:** Alec J Christensen, Emily K Latch, Michelle Carstensen, Travis Seaborn

**Affiliations:** School of Natural Resources, North Dakota State University, Fargo, ND 58102, United States; Department of Biological Sciences, University of Wisconsin-Milwaukee, Milwaukee, WI 53211, United States; Minnesota Department of Natural Resources, Wildlife Health Program, Forest Lake, MN 55025, United States; School of Natural Resources, North Dakota State University, Fargo, ND 58102, United States

**Keywords:** SNP panel, pedigree, relatedness, parentage, *Odocoileus virginianus*

## Abstract

Single nucleotide polymorphism (SNP) arrays have become increasingly popular due to their affordability, commercial availability, statistical power, and reproducibility. These arrays are being developed commercially for a wide range of species in various density formats. In this study, we evaluated the ability of commercially available medium-density (72,732 SNPs) and high-density SNP (702,183 SNPs) arrays for white-tailed deer (*Odocoileus virginianus*) to accurately identify known genetically related individuals within a wild population. We also assessed the impact of SNP filtering thresholds on relatedness analyses and compared the performance of 4 common relatedness software: KING, COLONY, Sequoia, and COANCESTRY, on these known related pairs. Our analysis revealed that the medium-density array exhibited greater tolerance to filtering and lower sensitivity to bioinformatic pipelines, making it a favorable balance between cost, computational time, and statistical power for analyses such as population structure. Additionally, we found that reducing missing data, specifically by using a subset of 600 loci with no missing data, combined with the relatedness estimator Sequoia (which allows the inclusion of life history data), yielded the most computationally efficient and accurate results. These findings offer valuable insights into the optimal SNP array size, appropriate filtering thresholds, and the most effective genetic relatedness methods for wildlife population studies.

## Graphical Abstract

**Graphical Abstract:**
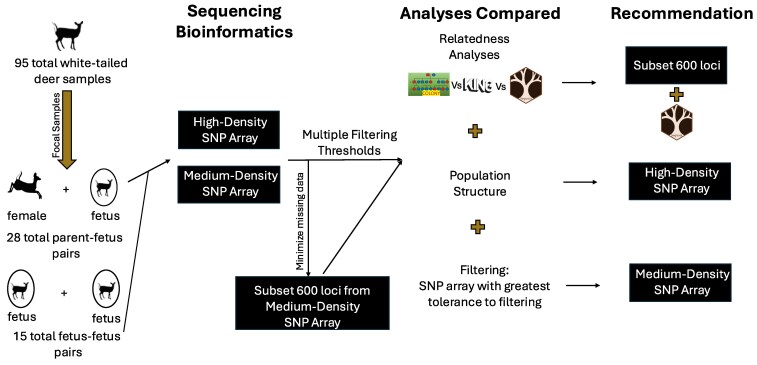

## Introduction

The genomic era has led to an increase in the accessibility of genome-wide data across a diverse range of taxa (Von Thaden et al. 2020). Despite these advancements, whole-genome sequencing remains cost-prohibitive for many studies. As a result, single nucleotide polymorphism (SNP) arrays have gained popularity. These arrays are widely used in animal genetic research due to their affordability and commercial availability (Matukumalli et al. 2009; Seabury et al. 2020; Zhang et al. 2020; Carrier et al. 2022). Designed to offer comprehensive genome coverage, SNP arrays allow the assessment of genetic metrics including population structure, inbreeding, and relatedness (Carrier et al. 2022). SNP arrays are available at varying densities, densities referring to the number of SNPs on the array, and have emerged as essential resources in genomic research. Although the classification of density size varies in the literature, a low-density array may broadly be defined as having fewer than 1,000 SNPs, a medium-density array as between 1,000 and 100,000 SNPs, and a high-density array as over 100,000 SNPs (Hagen et al. 2013; Vallejo et al. 2018). SNP arrays of different densities each present advantages and disadvantages, requiring an evaluation of tradeoffs between genomic resolution, cost, and computational resources. High-density arrays provide more detailed genetic resolution but come at a higher cost, being upwards of 2.5 times the cost of medium-density arrays per sample, and require significantly longer computational times and resources. However, medium-density arrays offer a cost-effective solution that balances genomic coverage and accuracy (Carrier et al. 2022). Studies comparing these 2 array types indicate that high-density arrays can improve the accuracy of genomic estimates, such as parentage analyses, population size, and inbreeding estimation (VanRaden et al. 2011; Su et al. 2012; Carrier et al. 2022). Depending on the analysis, high-density arrays may not always be preferable, as missing data and array-specific attributes can affect performance across applications. As SNP arrays continue to evolve, their applications extend beyond basic population genetics to broader questions in wildlife conservation and management, including answering questions centered around population dynamics and determining relatedness among individuals (Carranza et al. 2024; Thavornkanlapachai et al. 2024).

Genomic tools, particularly SNPs, are increasingly applied to address questions in wildlife conservation and management, including questions about genetic relationships among individuals and population structure (Hohenlohe et al. 2021). Identifying specific categories of relatedness, such as parent-offspring and sibling-sibling relationships, provides essential insights into genetic health, population structure, and broader population genomics, thereby informing conservation efforts and wildlife management (Schmidt et al. 2024). For example, in reintroductions, individuals sourced from a single population may exhibit some degree of relatedness, which can influence founding genetic diversity and reintroduction success (De Woody 2005; Rhodes and Latch 2010). Quantifying genetic relationships is especially valuable for species with less pronounced social structures, where kinship may not be readily apparent. Similarly, investigating genetic structure within social groups can address population-specific challenges, such as seasonal demographic shifts (Dobson et al. 1997; Comer et al. 2005; De Woody 2005; Latch and Rhodes 2006), or support disease management strategies (Grear et al. 2010; Magle et al. 2013) where agencies may target social groups to reduce disease transmission and prevalence (Prentice et al. 2019; Miguel et al. 2020). In disease ecology, understanding relatedness can enhance contact network analyses and facilitate the detection of kinship patterns, offering a deeper understanding of infectious disease dynamics (Blanchong et al. 2016).

Several methods exist for estimating genetic relatedness using SNP data, including KING (Manichaikul et al. 2010), COLONY (Jones and Wang 2010), Sequoia (Huisman 2017), and COANCESTRY (Wang 2011). These methods employ diverse genetic models and approaches to relatedness estimation, which can yield varying results (Hauser et al. 2022). KING implements a rapid algorithm for inferring relationships by estimating kinship coefficients and accounting for unknown population substructure, providing reliable results even for millions of individuals in just minutes (Manichaikul et al. 2010). COLONY uses full-pedigree likelihood methods to infer sibship and parentage among individuals. It is applicable to both diploid and haplodiploid species and supports both dominant and codominant markers while estimating genotyping errors (Jones and Wang 2010). Sequoia employs a heuristic hill-climbing algorithm to construct multigenerational pedigrees, clustering half-siblings, assigning parents, and identifying grandparents based on data from hundreds or thousands of SNPs (Huisman 2017). Sequoia also allows the ability to insert life-history data, allowing only pairs that are biologically feasible to be matched. Meanwhile, COANCESTRY calculates relatedness and inbreeding coefficients using a variety of estimators and a built-in simulation module to compare results, test group differences, and assess confidence intervals through bootstrapping and permutations (Wang 2011). As studies increasingly focus on relatedness and social structures to guide conservation and management, examining the tradeoffs between array density and accuracy of relatedness estimates will provide insights into the implications of array size selection, since the accuracy of these metrics could be influenced by array density.

Recently, Thermo Fisher Axiom genotyping arrays, commercially developed for a large number of species, became available in medium-density and high-density formats for white-tailed deer (*Odocoileus virginianus*) (Navarro et al. 2026). While these arrays can generate large amounts of genetic data that could inform deer management, their accuracy in identifying genetically related pairs has yet to be fully evaluated. White-tailed deer offspring are highly philopatric, exhibiting low dispersal rates and establishing home ranges that overlap with their mother; this pattern resembles petals on a rose and has been termed the “rose-petal hypothesis” (Porter et al. 1991). Because of the rose-petal structure and the role of female demography in driving population trends, management strategies often prioritize females. Targeting female social groups can effectively reduce population density and stabilize disease prevalence (McCullough 1999; Ueno et al. 2010; Manjerovic et al. 2014). This focus can have a large impact on reducing overall kinship within a population, which is effective in preserving genetic diversity and limiting inbreeding (Fernandez and Toro 1999; Sonesson and Meuwissen 2001; Giglio et al. 2016). One approach to reduce kinship is culling, also known as selective agency removal, which is a strategy used in certain wildlife disease management scenarios (e.g. bovine tuberculosis, chronic wasting disease [CWD]) to target social groups and reduce disease transmission and prevalence (Carstensen and DonCarlos 2011; Varga et al. 2022). These kinship-based strategies are most effective when genetic relatedness is known, allowing for informed agency removal and other targeted management in wild populations. Accurate estimates of relationships are essential to ensure that efforts to reduce kinship are both targeted and effective, for example, by evaluating whether disease-focused management strategies are successfully targeting and removing high-risk social groups that contribute most to disease transmission and spread. Accurate identification of related individuals also enables pedigree-informed approaches such as close-kin mark-recapture, which can provide reliable estimates of population size and structure (Bravington et al. 2016). In addition to assessing relatedness, SNP arrays may also offer the resolution needed to detect patterns of neutral population structure, which provides understanding of gene flow and genetic connectivity across the landscape (Helyar et al. 2011). With the increasing availability of commercial SNP arrays, evaluating whether medium-density or high-density SNP arrays provide a higher resolution for identifying related pairs and detecting population structure will help guide future studies and inform the effectiveness of these management strategies in selecting related individuals.

This project aimed to evaluate the effectiveness of commercial SNP arrays in assessing genetic relationships using known genetically related pairs. While medium-density and high-density arrays have been compared in domestic animals, such comparisons are relatively rare in wild populations, in part because there are few commercial genotyping panels available for wild species. Additionally, wild species are often expected to have higher levels of genetic variation, which may influence their performance differently compared to arrays used in domestic species. This study will address the following objectives: (i) evaluate the influence of filtering for missing data on relatedness estimation; (ii) investigate whether a medium-density or high-density SNP array is more effective at revealing population structure; (iii) determine whether a medium-density or high-density SNP array is more effective at identifying genetic relationships in wild white-tailed deer; and (iv) compare the medium-density and high-density arrays across varying genetic relatedness methods, (KING, COLONY, Sequoia, and COANCESTRY) to see how differences in analytical approaches affect the comparison. In addition to the 2-array datasets, we created a third dataset (600-loci) from the medium-density array with no missing genotype data to align with the suggestions of Sequoia (Huisman 2024) for our third and fourth objectives. The results of this study will provide insight into the optimal number of genomic markers, appropriate filtering thresholds, and the most accurate genetic relatedness method for the Axiom medium-density and high-density white-tailed deer SNP arrays. Lastly, this study will offer significant insights for guiding future genomic and genetic management efforts in other wild populations.

## Methods

### Study population

The Minnesota Department of Natural Resources provided samples as part of their CWD management efforts. The dataset consisted of 95 individuals, including 1 duplicate sample to check for genotyping accuracy. Samples were collected in 2017 and 2019 to 2023 through agency culling (92 individuals and 1 duplicate) and shooting permits (3 individuals) (Supplementary Fig. 1). The dataset includes 46 adults (42 females and 4 males), 10 yearlings (7 females and 3 males), 11 fawns (6 females and 5 males), and 28 fetuses (12 females and 16 males), including 28 known parent-offspring pairs (female-fetus) and 15 known full-sibling pairs (fetus-fetus). Hereafter, female–fetus pairs are referred to as parent–offspring, and fetus–fetus pairs as full-siblings.

### Impacts of filtering and quality control

Muscle and lymph node samples were collected and stored at −20 °C until DNA extraction. DNA was extracted from a 2 mm^3^ piece of each sample using DNeasy Blood and Tissue Kits (Qiagen, Hilden, Germany) and the animal tissue protocol, with 0.4 mg of proteinase K added and an overnight incubation during the initial lysis step. Samples from the same family were not extracted in the same batch, and negative blank extraction controls were included in each batch of extractions. DNA concentration and quality were assessed using both a fluorescent Qubit BR assay and a Nanodrop UV-Vis spectrophotometer. The A260/280 ratios for all samples ranged from 1.7 to 2.2, and the A260/230 ratio was >1.4. The DNA extracts were then normalized to a concentration of 30 ng/µL by drying down a total of 750 ng of DNA and rehydrating with 25 µL of sterile molecular-grade water. Libraries were prepared using the Axiom 2.0 Assay workflow by amplifying the total genomic DNA, fragmenting it into smaller uniform pieces, and preparing a hybridization tray to sandwich to the array plate. The hybridization and genotyping (wash/stain/scan steps) were performed using an Applied Biosystems GeneTitan Multi-channel microarray instrument and manufacturer's protocols at North American Genomics (Decatur, GA). One sample was included as a duplicate to verify the accuracy of genotyping calls.

We genotyped our white-tailed deer samples using the Axiom OVSNP600 and Axiom OVSNP60 genotyping arrays from Thermo Fisher Scientific (Thermo Fisher Sicentific, Waltham, Massachusetts, United States; referred to as high-density (OVSNP600) and medium-density (OVSNP60) arrays henceforth). The high-density array was designed from resequencing data and contains 702,183 SNPs. These SNPs were selected to provide broad genomic representation and are evenly distributed across the scaffolds of the white-tailed deer reference genome. Of the SNPs on the high-density array, 517,261 were classified as “Best and Recommended” in an initial screening from 480 free-ranging white-tailed deer collected across 15 states in the eastern United States (Navarro et al. 2026); where “Best and Recommended” refers to SNP probe conversion types that yield reliable, high-quality genotype calls (polyhighres, monohighres, and nominorhom) while excluding problematic classes such as off-target variants. The medium-density array contains 72,732 SNPs and was developed by subsampling from the “Best and Recommended” SNPs on the high-density array (Navarro et al. 2026). Redundancy is built into these arrays in the same way as most SNP genotyping arrays, by using multiple probes for each SNP to improve signal consistency, reduce false positives, increase genotyping accuracy, and facilitate error detection.

Genotype data was output into 2 variant call files (VCFs) including all 95 individuals (with 1 duplicate) for the medium-density (67,457 SNPs) and high-density (600,866 SNPs) arrays. Using the VCF files for the medium and high-density arrays, we applied additional filtering to assess how sensitive relatedness estimates are to missing data. We imported both the medium-density and high-density VCFs into PLINK v2.0 (Chang et al. 2015) for additional filtering and initial relatedness analyses. Using the –geno (loci) and –mind (individual) flags in PLINK, we filtered genotype data to exclude SNPs or individuals with high proportions of missing data. The filtering process was conducted in 2 steps: first by removing SNPs with excessive missingness using the –geno command, then by excluding individuals with a high number of missing loci using the –mind flag. For both arrays, we tested 9 filter combinations, with –geno thresholds of 15%, 20%, and 25% and –mind thresholds of 10%, 15%, and 20% (Table 1). This approach allowed us to evaluate the impact of different levels of missing data on the accuracy of identifying known related pairs. Through our analyses and discussions with the developer of Sequoia, we learned that using a lower density of SNPs can yield greater accuracy within Sequoia's framework (Huisman 2017; Huisman 2024). Using this insight, we selected a random subset of 600 loci from the medium-density array, with no missing data. This subset will hereafter be referred to as “600-loci”. In total, we analyzed 3 data sets: 600-loci, medium-density, and high-density.

**Table 1.** Filtering thresholds for medium-density and high-density arrays, set using the –geno and –mind flags in PLINK.

SNP missingness (−geno)	Individual missingness (−mind)
15%	10%
15%	15%
15%	20%
20%	10%
20%	15%
20%	20%
25%	10%
25%	15%
25%	20%

The –geno flag removed SNPs with excessive missing data, while the –mind flag excluded individuals with a high proportion of missing SNPs.

For each of our 9 filter combinations, we calculated the number of excluded individuals and assessed the accuracy of parent-offspring identification. This specifically allowed us to balance the tradeoff between retaining the maximum number of SNPs and individuals while ensuring high confidence in parent-offspring identification. For each combination, we calculated the number of excluded individuals, number of retained SNPs, number of known parent-offspring pairs identified with Sequoia (see below) and the F1 score; which is the harmonic mean of precision and recall and is derived from true positives, false positives, and false negatives (Tharwat 2021). Although the number of true positives is known because it reflects our known parent–offspring pairs, the true number of false negatives remains unknown, as some individuals in this sampled wild population with unknown kinship could be full-sibling or parent-offspring pairs. By comparing these results, we aimed to identify optimal filtering thresholds that maintained dataset quality without compromising the accuracy of relatedness assignments.

### Kinship analyses

#### PLINK-KING

To assess relatedness among individuals within the arrays, we used the –make-king flag within PLINK, which applies the KING-robust kinship estimator (Manichaikul et al. 2010). This method allowed us to identify and classify relationships into duplicate or monozygotic twin, first-degree, second-degree, and third-degree relationships. Relationship classifications were based on the following thresholds: >0.354 for duplicate or monozygotic twin pairs, [0.177, 0.354] for first-degree relatives, [0.0884, 0.177] for second-degree relatives, [0.0442, 0.0884] for third-degree relatives, and [<0, 0.0442] for distant to unrelated individuals (Chang et al. 2015). We leveraged having known parent-offspring and full-sibling relationships to evaluate the accuracy of finding first-degree related pairs.

#### Sequoia

We used the Sequoia package v2.11.4 (Huisman 2017) in R v4.4.2 (R Core Team 2024) to assess the accuracy of identifying parent-offspring and full-sibling relatedness among individuals within the 3 datasets. By incorporating the *agepriors* argument, a set of probability ratios based on age differences (Huisman 2024), along with known life history data such as sex, birth year, and death year, we aimed to enhance the accuracy of parent-offspring pair assignments. For most individuals, we had known ages, which allowed us to calculate their birth year by subtracting their age from their death year. For individuals without a known age, we utilized the birth year minimum and birth year maximum options within Sequoia. For individuals without known ages, we assigned birth year ranges based on recorded age classes: adult, yearling, fawn, and fetus. For adults, the birth year minimum was set to 15 yr before the death year, and the birth year maximum was 2 yr before the death year. For yearlings, the birth year minimum was 2 yr before the death year, and the birth year maximum was 1 yr before the death year. For fawns, the birth year minimum was 1 yr before the death year, and the birth year maximum was also 1 yr before the death year. For fetuses, both the birth year minimum and birth year maximum corresponded to the death year (Supplementary Table 1). These ranges ensured that plausible parent-offspring relationships were accurately captured. The *ageprior* defaulting parameters were used, except for the minimum age of parent, that was set to 1. We then applied the *GetMaybeRel* function to the genotype and *ageprior* data. *GetMaybeRel* identifies any pairs that are likely to be first- or second-degree relatives (Huisman 2024). Using the “*Par”* module, we performed a parentage assignment to identify our parent-offspring pairs, where genotyped parents were matched to genotyped offspring. Default settings were used for both the “*Par*” and “*Ped*” modules, except for the error rate, which we adjusted to 0.175, for the medium-density and high-density arrays, to account for genotyping errors in the data, and the suggestion that the rate should be adjusted slightly higher than anticipated (Huisman 2024). The 600-loci dataset was adjusted to 0.07. These error rates allowed us to achieve consistent parent-offspring and full-sibling matches, which we leveraged. These error rates were determined based on the values that yielded the highest number of correctly identified known parent-offspring pairs across a range of tested values (0.01 to 0.25).

Due to the accuracy (see below), ability to insert life-history data, and relatively short computational time, we used Sequoia to gather relatedness estimates on our parent-offspring pairs on our 9 filter combinations for both the medium-density and high-density arrays. We chose the filtering combination that identified the most parent-offspring pairs while retaining the greatest number of SNPs and individuals. The determined filtering combination for both arrays was used in all downstream analyses.

#### COLONY

Our next relatedness analysis was conducted using COLONY v2.0.6.8 (Jones and Wang 2010), a software designed to assess parent-offspring, full-sibling, and half-sibling relationships. Parameters for COLONY consisted of the following: male and female polygamy (DeYoung et al. 2002), no inbreeding or cloning, long runs with full-likelihood (Wang 2012), medium precision, no sibship-scaling, no sibship size prior, a maternal probability set to 0.7, and paternal probability set to 0.2 (Fameli et al. 2025). For the high-density array, a short run was performed instead of a long run due to computational time constraints. A long run is 100 times longer than that of a short run (Jones and Wang 2010), which would have been over a month for our high-density dataset. Additionally, we included one known parent-offspring pair as a known maternal relationship. These parameters were chosen to optimize the detection of relationships while balancing computational time constraints.

#### COANCESTRY

For our final relatedness analysis method, we implemented the program COANCESTRY v1.0.1.11 (Wang 2011). COANCESTRY was used to compare pairwise relatedness estimates between our individuals. The program generated relatedness estimates using 7 estimators, including methods described by Queller and Goodnight (1989), Li et al. (1993), Ritland (1996), Lynch and Ritland (1999), and Wang (2002). Additionally, we employed the dyadic likelihood estimator from Milligan (2003) and the triadic likelihood estimator from Wang (2007). Parameters were set to assume unknown allele frequencies and 95% confidence intervals were obtained through bootstrapping. This involved resampling 100 loci and 100 reference individuals across the dataset (Wang 2011).

#### Comparison of relatedness methods

In total, we used 4 methods: PLINK-KING, Sequoia, COLONY, and COANCESTRY, to assess known parent-offspring and full-sibling pairs in our dataset. PLINK-KING, Sequoia, and COLONY were compared on their ability to identify the known parent-offspring and full-sibling pairs and for these 3 methods we calculated F1 scores across all datasets to evaluate performance. For all of these methods, we compared the performance of identifying parent-offspring and full-sibling pairs across the datasets, allowing us to evaluate the tradeoffs between these commonly used relatedness methods in determining parent-offspring and full-sibling pairs. In contrast, COANCESTRY was used to compare relatedness estimates across datasets using 7 different estimators. For COANCESTRY, we took the average and range of relatedness values for each estimator across the known parent-offspring and full-sibling pairs, providing additional context on the consistency and variability of relatedness estimates across our datasets.

### Population structure

To explore population structure, we employed a combination of principal component analysis (PCA) and Admixture analysis to identify genetic variation and evaluate potential differences among the datasets. We performed a PCA using the *dudi.pca* function in the ade4 R package (Dray and Dufour 2007). We wanted to explore population structure among our sampled individuals and assess variation among the data sets. A PCA offers a comprehensive overview of genetic variation without group constraints, which can obscure subtle patterns associated with specific factors. To investigate population structure further, we used the software Admixture v1.0.1.11 (Alexander et al. 2009) and assessed cross-validation scores for varying numbers of ancestral populations (*K* = 1 to *K* = 10). This approach allowed us to evaluate the optimal number of genetic clusters per SNP array and gain a deeper understanding of population structure among our individuals along with differences among our arrays, complementing the insights provided by PCA.

## Results

### Genomic datasets and quality control

**Fig. 1.**
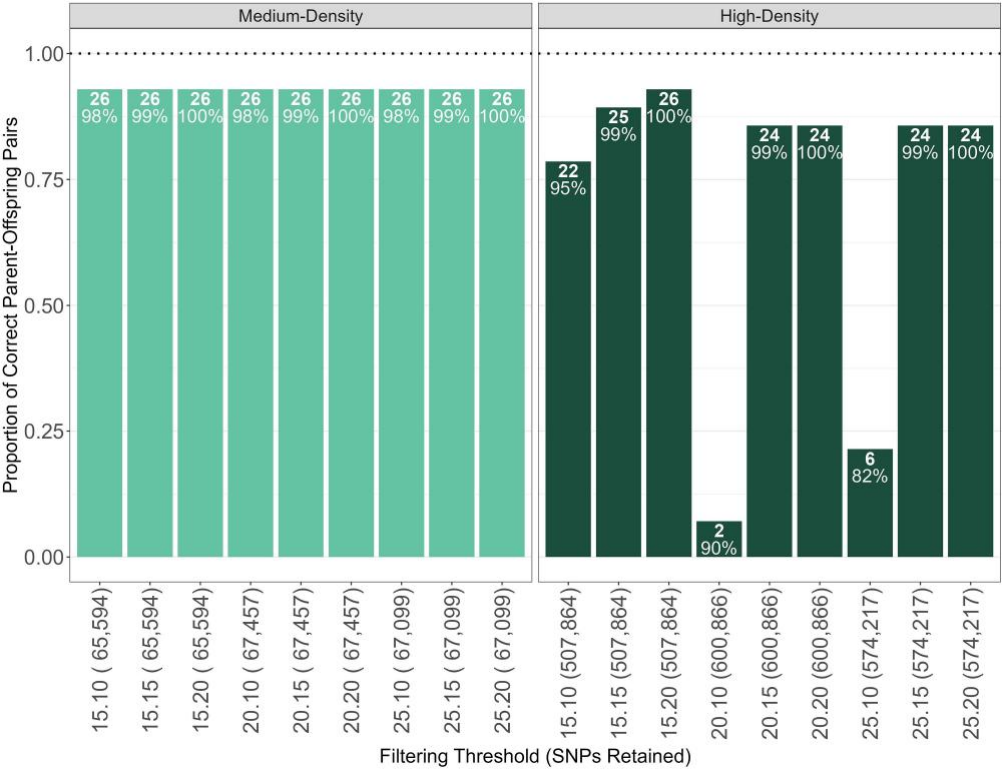
Sequoia results for known parent-offspring pairs across varying filtering thresholds in the medium-density and high-density arrays. The *x*-axis represents PLINK filtering thresholds, beginning with SNP removal based on missingness using the –*geno* command (15, 20, or 25%), followed by individual exclusion using the –*mind* command (10, 15, or 20%), and concluding with the number of retained SNPs in parentheses. Within each column, the top value indicates the number of correctly identified parent-offspring matches (out of *n* = 28 pairs), while the percentage below represents the proportion of individuals retained (out of *n*= 96 individuals).

We genotyped 96 white-tailed deer samples using the Axiom OVSNP60 and the Axiom OVSNP600 genotyping arrays from Thermo Fisher Scientific, resulting in 67,457 SNPs in the medium density-array, and 600,866 SNPs in the high-density array. The 9 filtering thresholds among the VCFs resulted in varying numbers of retained individuals, retained loci, and number of parent-offspring pairs identified (Fig. 1). We saw a greater variation in the high-density array across filtering thresholds than in the medium-density array. Similarly, F1 scores differed between panels: the medium-density array was consistently stable across filtering thresholds (0.73 to 0.77), whereas the high-density array showed greater variability (0.10 to 0.76; Table 2).

**Table 2.** Summary of relatedness estimates performance across filtering thresholds in the medium-density and high-density arrays.

Data set	Filtering threshold (−geno.–mind)	True positives	False positives^a^	False negatives	F1 statistic
Medium-density	15.10	26	14	2	0.77
Medium-density	15.15	26	15	2	0.75
Medium-density	15.20	26	17	2	0.73
Medium-density	20.10	26	14	2	0.77
Medium-density	20.15	26	14	2	0.77
Medium-density	20.20	26	17	2	0.73
Medium-density	25.10	26	14	2	0.77
Medium-density	25.15	26	15	2	0.75
Medium-density	25.20	26	17	2	0.73
High-density	15.10	22	12	6	0.71
High-density	15.15	25	13	3	0.76
High-density	15.20	26	15	2	0.75
High-density	20.10	22	9	26	0.10
High-density	20.15	25	13	4	0.74
High-density	20.20	26	16	4	0.71
High-density	25.10	2	9	22	0.28
High-density	25.15	24	13	4	0.74
High-density	25.20	24	16	4	0.71

Metrics include the number of true positives, false positives, and false negatives, along with calculated F1 statistics.

^a^Note that the true number of false positives is unknown, as this is a wild population and we cannot determine for certain relationships outside of our set of known related pairs.

We proceeded with our analyses using the following filtering thresholds: the medium-density array without any additional filtering as it retained the largest number of loci and individuals, and the high-density array filtered to remove loci with more than 15% missing data and individuals with more than 20% missing genotypes, retaining all individuals and 507,864 SNPs. Since the highest number of SNPs and the greatest proportion of known matches were identified under these filtering thresholds, we maintained them for all subsequent analyses.

After applying these thresholds, the final medium-density dataset contained an average of 3.2% missing genotypes per individual (range: 0.9% to 15.6%), while the high-density dataset contained 4.4% missing genotypes per individual on average (range: 1.2% to 17.2%). At the locus level, the average of missing data was 3.2% (range: 0% to 93.0%) for the medium-density panel and 4.4% (range: 0% to 14.6%) for the high-density panel.

### Kinship analyses

#### PLINK-KING

**Fig. 2.**
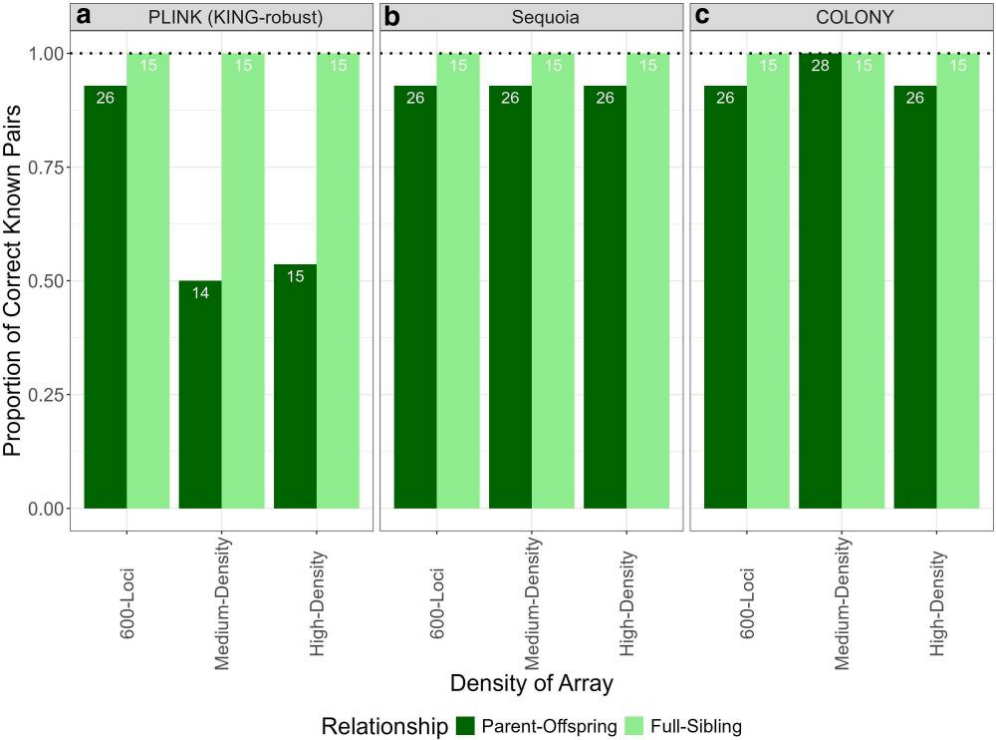
Comparison of relatedness analysis methods in identifying known genetically related parent-offspring pairs (*n* = 28) and full-sibling pairs (*n* = 15) across 3 datasets: 600-loci, medium-density, and high-density. The analyses include a) KING-robust (implemented in PLINK), b) Sequoia, and c) COLONY. Numbers within each column represent the count of correctly identified known pairs. Within Sequoia, the module “*Par*” was used to find the full-sibling pairs in the medium-density and high-density array, due to the “*Ped*” module failing to find full-sibling pairs in the medium-density and high-density array. “*Par*” is a parental assessment and “*Ped*” is a pedigree assessment (see Supplementary Fig. 2 for “*Par*” vs “*Ped*” comparison).

Using KING within PLINK the 600-loci dataset found 15 out of the 15 (100%) full-sibling pairs and 26 out of the 28 (93%) parent-offspring pairs, resulting in an F1 score of 0.83. For the medium-density array, 15 out of the 15 (100%) full-sibling pairs were identified, while only 14 out of the 28 (50%) parent-offspring pairs were found, yielding an F1 statistic of 0.78. For the high-density array, 15 out of the 15 full-sibling pairs and 15 out of the 28 (54%) parent-offspring pairs were found, resulting in an F1 statistic of 0.67 (Fig. 2a; Table 3).

**Table 3.** Summary of relatedness estimate performance across methods (Sequoia, PLINK, and Colony) and SNP datasets.

Method	Data set	True positives	False positives^a^	False negatives	F1 statistic
Sequoia	600-Loci	41	16	2	0.82
PLINK	600-Loci	41	15	2	0.83
COLONY	600-Loci	41	11	2	0.86
Sequoia	Medium-density	26 (+15)	17	2	0.73
PLINK	Medium-density	41	21	2	0.78
COLONY	Medium-density	43	11	0	0.89
Sequoia	High-density	26 (+15)	15	2	0.74
PLINK	High-density	41	38	2	0.67
COLONY	High-density	41	10	2	0.87

Metrics include the number of true positives, false positives, and false negatives, along with calculated F1 statistics. Values in parentheses (e.g. 26 [+15]) indicate full-sibling relationships identified in the Sequoia parentage assesment (“*Par”*) that were not detected in the pedigree assesment (“*Ped*”), as explained in the Methods.

^a^Note that the true number of false positives is unknown, as this is a wild population and we cannot determine for certain relationships outside of our set of known related pairs.

#### Sequoia

Our Sequoia analyses for all our datasets demonstrated high accuracy in identifying parent-offspring relationships when using the “*Pa*r” module within the *GetMaybeRel* function. The 600-loci dataset found 15 out of the 15 (100%) full-sibling pairs and 26 out of the 28 (93%) parent-offspring pairs, corresponding to an F1 score of 0.82. For the medium-density array, 0 out of the 15 (0%) full-sibling pairs were identified, while finding 26 out of the 28 (93%) parent-offspring pairs. For the high-density array, we found 0 out of the 15 full-sibling pairs, while finding 26 out of the 28 (93%) parent-offspring pairs, resulting in an F1 score of 0.73 when excluding the full-sibling pairs. Interestingly, Sequoia was unable to identify full-sibling pairs in the medium-density and high-density arrays with the “*Ped*” module. However, when the medium-density and high-density arrays were run using the “*Par*” module and no life history data, all 15 full-sibling pairs were identified as first-degree relationships (Fig. 2b; Table 3; Supplementary Fig. 2). To further investigate why full-sibling pairs were not identified using the “*Ped”* module, we conducted a duplicate check in Sequoia for both arrays. This analysis was performed independently of the *GetMaybeRel* function, which does not include a built-in duplicate detection tool. Using Sequoia's *sequoia* function with the “*dup*” module, 7 of the 15 expected full-sibling pairs in the 600-loci dataset, 2 of 15 in the medium-density array, and 1 of 15 in the high-density array were incorrectly flagged as duplicates.

#### COLONY

COLONY effectively identified parent-offspring and full-sibling relationships across all our datasets. The 600-loci dataset found 15 out of the 15 (100%) full-sibling pairs and 26 out of the 28 (93%) parent-offspring pairs, resulting in an F1 Score of 0.86. These 2 parent-offspring pairs were the same pairs that were not identified with KING-PLINK and Sequoia. For the medium-density array, 15 out of the 15 (100%) full-sibling pairs were identified, and 28 out of the 28 (100%) parent-offspring pairs were found, corresponding to a F1 score of 0.89. For the high-density array, we found 15 out of the 15 full-sibling pairs and 26 out of the 28 (93%) parent-offspring pairs, yielding a F1 score of 0.87 (Fig. 2c; Table 3).

#### COANCESTRY

**Fig. 3.**
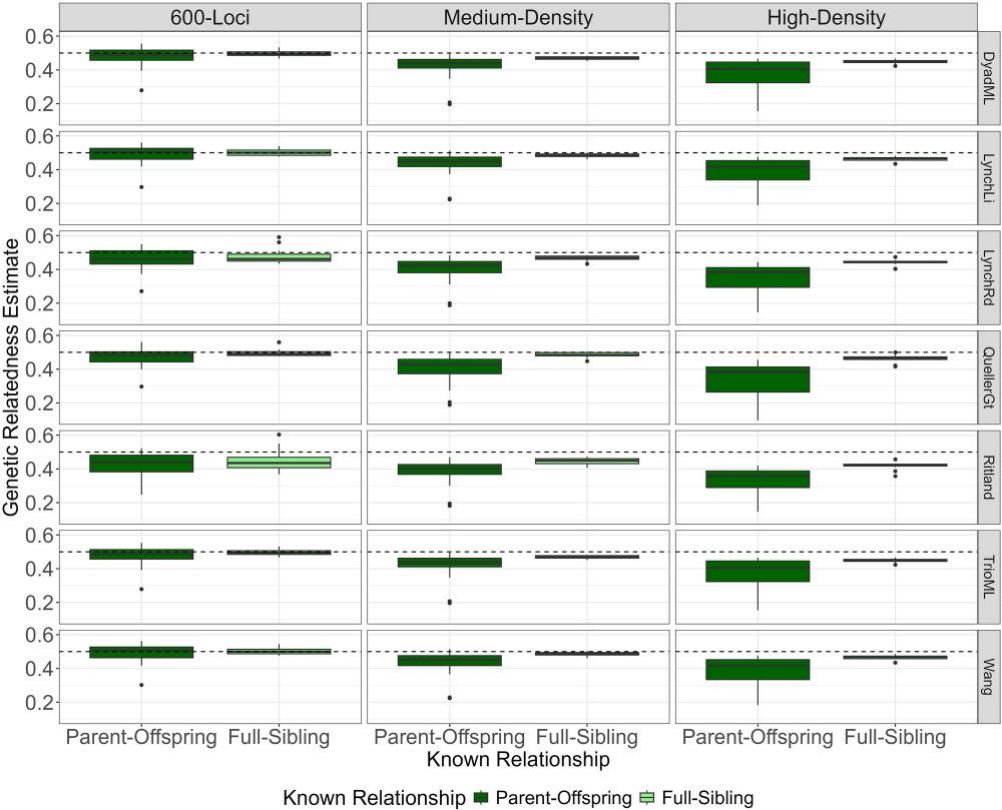
COANCESTRY results for known parent-offspring and full-sibling pairs across 3 datasets: 600-loci dataset, medium-density array, and high-density array. Analyses were conducted using 7 relatedness estimators: Dyadic Likelihood Estimator (DyadML), Li (LynchLI), Lynch & Ritland (LynchRd), Ritland, Milligan (TrioML), and Wang.

COANCESTRY showed similar results among the different estimators and the different datasets. The parent-offspring pairs showed a wider range of calculated relatedness estimates across the different estimators than the full-sibling pairs (Fig. 3). Across our 3 datasets, we averaged the relatedness estimates from 7 estimators and the range of the average values for each relationship category: parent-offspring, problematic parent-offspring (2 pairs rarely identified in previous analyses), and full-sibling pairs. The 600-loci dataset produced the highest estimates, with averages ranging from 0.44 to 0.50 for parent-offspring, 0.21 to 0.31 for the problematic parent-offspring pairs, and 0.45 to 0.51 for full-sibling pairs. The medium-density dataset showed slightly lower values: 0.40 to 0.45 (parent-offspring), 0.19 to 0.23 (problematic parent-offspring), and 0.47 to 0.49 (full-sibling). The high-density dataset yielded the lowest estimates, ranging from 0.35 to 0.40 (parent-offspring), 0.14 to 0.21 (problematic parent-offspring), and 0.42 to 0.47 (full-sibling). Overall, the 600-loci dataset provided estimates closest to the theoretical expectation for first-degree relatives (0.50). Ranges for all relatedness estimators across datasets are presented in Supplementary Table 2.

### Population structure

**Fig. 4.**
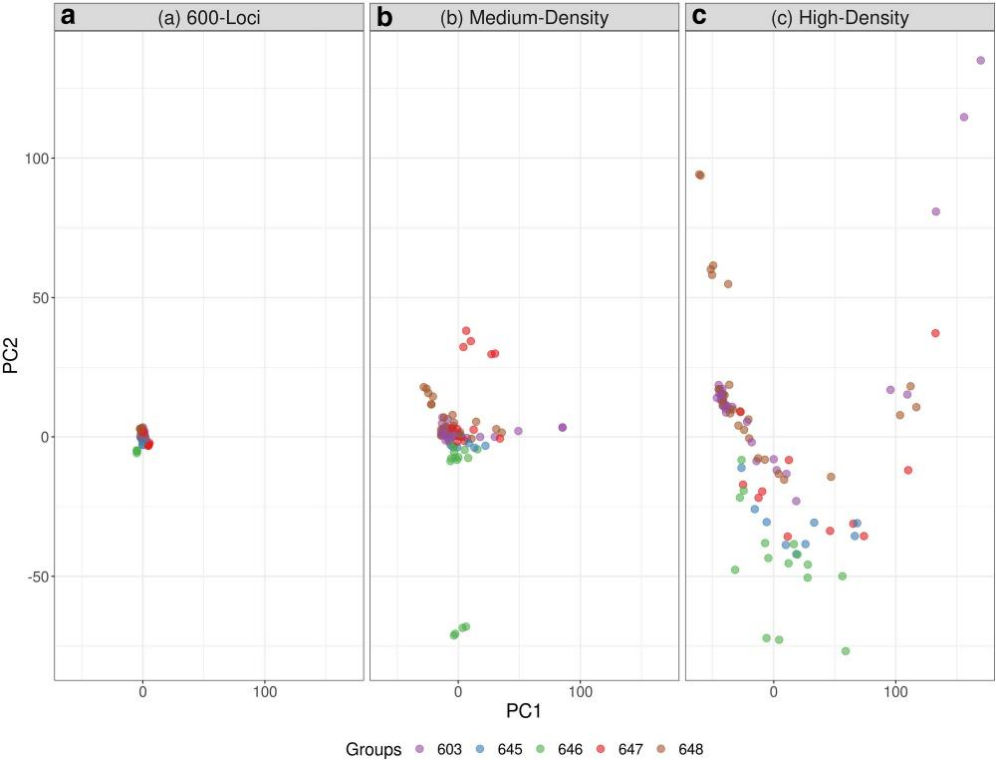
Principal component analysis (PCA) of individual genotypes across 3 datasets: a) 600-loci dataset, explaining a combined 6.4% of total variance between PC1 and PC2; b) medium-density array, explaining a combined 5.6% of total variance between PC1 and PC2; c) high-density array explaining a combined 6.4% of total variance between PC1 and PC2. The colors of the points identify the 5 management areas for white-tailed deer in Minnesota.

Our PCA results indicated weak population structure, with no clear clustering observed across any of the arrays (Fig. 4). However, as the number of SNPs increased, we observed a trend toward increased resolution. At the 600-loci level, the samples were clustered tightly together, suggesting no structure. With the medium-density array, although there was still no structure, the samples began to spread out, showing a potential signal of differentiation. The high-density array showed the samples were more widely dispersed, though there was no clear clustering by management area. Admixture analysis supported these observations, with the 600-loci and medium-density datasets indicating the strongest support for a single ancestral population (*K* = 1) (Supplementary Fig. 2), while the high-density array suggested a marginal signal for 2 ancestral populations (*K* = 2) (Supplementary Fig. 3). Although *K* = 2 is identified as the top model, this value can misrepresent the true number of populations, either underestimating or overestimating them (Janes et al. 2017). These results suggest that higher SNP density increases the statistical resolution of population structure, with the high-density array providing more detailed and refined insights. It is important to note the limited number of samples used in the analysis, as well as their origin; the majority of these individuals were agency-culled and sampled within a small geographic area of southeast Minnesota (Supplementary Fig. 1).

### Comparison of arrays

In comparison, there are tradeoffs for the medium-density and high-density arrays. The medium-density array is a cost-effective option, being nearly half the price of the high-density array, and demonstrating a greater tolerance to a variety of filtering thresholds. Computationally, the medium-density array had faster runtimes for all methods evaluated, requiring less than half the time for the majority of methods. For instance, runtimes for COLONY were approximately 11 d for the medium-density array compared to 31 d for the high-density array. For related analyses, individual samples could be genotyped for either array and be filtered down to a low amount of loci, as we did with 600 loci, to make the analyses more computationally sufficient and for some methods such as PLINK-KING more accurate. In contrast, the high-density array is more expensive and less tolerant to stringent filtering, resulting in greater loss of SNPs and individuals. Although the high-density offers potentially higher resolution for analyses such as population structure, it does come with higher computational demands. Overall, the medium-density array is sufficient for relatedness estimates and is especially efficient when analyses can be performed on subsets of loci (Table 4).

**Table 4.** Tradeoffs between medium-density and high-density SNP arrays, including cost, tolerance to filtering, informativeness, and computational demands.

Category	Medium-density array	High-density array
Approximate Cost per Sample	∼$60/sample	∼$120/sample
Filtering Tolerance	Greater tolerance to filtering; retains more individuals/SNPs across thresholds	Lower tolerance to filtering; more SNPs and individuals removed under stringent thresholds
Analytical Informativeness	Sufficient for relatedness and pedigree reconstruction	Likely more informative for analyses requiring fine-scale resolution, e.g. population structure
Computational Demands	Lower computational burden, faster runtimes	Much higher computational burden, slower runtimes
Example Runtimes by Method		
PLINK (KING)	<1 min	<1 min
Sequoia	<10 min	<40 min
COLONY	∼16,060 min (≈11 d)	∼44,880 min (≈31 d)
COANCESTRY	∼458 min (≈7 h)	∼6,431 min (≈4 d)
Runtimes for 600-Loci	PLINK (KING): <1 min; Sequoia: <1 min; COLONY: ∼210 min; COANCESTRY: <5 min	PLINK (KING): <1 min; Sequoia: <1 min; COLONY: ∼210 min; COANCESTRY: <5 min

Estimated runtime (in minutes) for each dataset (*n* = 96) is shown for PLINK (KING), COLONY, Sequoia, COANCESTRY. All runs were conducted using the parameters described in the Methods. For COLONY, the high-density array is reported as a long-run estimate rather than the short run used in our study to save time (a long run is approximately 100 times longer than a short run; Jones and Wang 2010). The COLONY medium-density and high-density runtimes were timed to one-eleventh of completion, and the reported values were extrapolated based on the approximate elapsed time.

## Discussion

We examined the effectiveness of commercial medium-density and high-density SNP arrays, as well as the potential of a low-density SNP array, on white-tailed deer in assessing genetic relationships using known genetically related pairs. Determining relatedness among array density has been compared in various domestic animals (Lopes et al. 2013; Ventura et al. 2016); however, comparison in wild animals has been relatively rare up to this point. First, through filtering experiments, we determined that removing loci and individuals with a high proportion of missing data is crucial in the high-density array and certain filtering thresholds can provide a more accurate representation of relatedness matches. Secondly, we found the high-density SNP array showed trends suggesting it could potentially provide greater statistical resolution in detecting population structure, giving a more detailed and refined insight. Third, our analyses determined that the 600-loci dataset was able to more reliably identify known parent-offspring and full-sibling pairs in our white-tailed deer samples. Lastly, we found Sequoia to be a computationally efficient approach that leverages life history data to support accurate genetic relatedness estimates. This information can guide managers in proper array selection, data filtering, and relatedness estimate selection, not only for white-tailed deer but for other wildlife as well.

Filtering thresholds demonstrated clear differences in SNP retention and individual inclusion between the medium-density and high-density arrays which may be important for making management decisions. The medium-density array exhibited consistency and accuracy across all filtering thresholds, retaining the majority of individuals and SNPs for analyses. A similar trend in the impact of filtering has been documented in more in-depth modeling studies (Kling and Tillmar 2019; Tillmar and Kling 2025). The medium-density array's ability to deliver reliable kinship analyses despite missing loci and minor data quality issues positions it as a promising, cost-efficient option for future studies in population genetics and management. In our study, the high-density array cost roughly twice as much as the medium-density array. Specifically, the medium-density array cost approximately $45 per sample for the array plus $15 for genotyping, totaling $60 per sample, whereas the high-density array cost approximately $105 per sample plus $15 for genotyping, totaling $120 per sample. In contrast to the medium-density array, the high-density array showed greater sensitivity to filtering thresholds. This result is similar to other studies that found missing data to be problematic when determining relatedness (Dodds et al 2015; Attard et al. 2018; Gervais et al. 2019). While the high-density array offers a larger number of SNPs for finer-resolution analyses, its vulnerability to missing data necessitates careful threshold selection to avoid excessive data loss, emphasizing the tradeoff between resolution and SNP retention. Our results align with Chang et al. (2018), which investigated filtering thresholds across various array sizes, finding that high-density arrays were more sensitive to filtering, resulting in greater performance declines compared to medium-density arrays. It is important to note studies have suggested SNP filtering strategies should be selected based on the specific species (Noda et al. 2025), and estimator method (Speed and Balding 2015; Huisman 2017). Future research aiming for more comprehensive genomic coverage could leverage the enhanced resolution of the high-density array, provided that additional measures are taken to address data quality and filtering challenges. In wildlife systems, studies exploring relatedness should prioritize minimizing missing data regardless of array size.

We found that the 600-loci dataset provided the most reliable relatedness estimates, while the high-density array offered better resolution for population structure. The 600-loci dataset was consistent across relatedness estimators, demonstrating accuracy and reliability in identifying full-sibling and parent-offspring pairs. These findings align with previous studies showing that low-density datasets are effective for relatedness analysis (Huisman 2017; Attard et al. 2018). The 600-loci dataset performed as well as or better than both the medium-density and high-density array, except for when using COLONY, where the medium-density array excelled. However, the 600-loci dataset's strength in relatedness analysis came at the cost of lower population structure resolution potential. The medium-density array showed variability across estimators but excelled in COLONY, identifying all known pairs. This supports Speed and Balding (2015), who noted that estimator performance depends on dataset characteristics. For other estimators, its performance was comparable to the 600-loci and high-density arrays. Notably, medium-density arrays have been found to be particularly effective for detecting distant relationships, such as third to seventh-degree relatives (Tillmar and Kling 2025). Additionally, the medium-density array provided moderate resolution for population structure analysis. The high-density array yielded inconsistent results in PLINK and never outperformed the 600-loci or medium-density datasets in any relatedness analysis. Computationally, it posed challenges for most estimators outside of PLINK but remained reliable for identifying full-sibling and parent-offspring pairs in COLONY. While studies comparing SNP array densities for relatedness in wildlife are rare, our findings align with research demonstrating the effectiveness of low-density SNP arrays in relatedness analysis (Sellars et al. 2014; Huisman 2017; Silva et al. 2022; Kriaridou et al. 2023). In contrast, the high-density array showed greater potential for population structure resolution. Although *K* = 2 can misrepresent the true number of populations (Janes et al. 2017), this outcome can result from factors such as extensive gene flow between groups (Latch et al. 2006; Waples and Gaggiotti 2006), the presence of close relatives (vonHoldt et al. 2010; Rodriguez-Ramilo and Wang 2012), and limited sample sizes (Waples and Gaggiotti 2006). Future research should explore population structure at broader geographic scales where differentiation is expected.

Empirical estimates of genetic relatedness among individuals often deviate from theoretical expectations based on Mendelian inheritance and identity by descent, which reflects the probability that 2 alleles, one randomly drawn from each of 2 individuals, are inherited from a common ancestor (Ballou 1983; Lacy 1995). In the absence of inbreeding, the expected relatedness coefficient is 0.5 for both parent-offspring and full-sibling pairs, indicating that they share approximately 50% of their genome. However, observed estimates typically fluctuate around this value and may fall either above or below the 0.5 threshold (Powell et al. 2010; Speed and Balding 2015; Galla et al. 2020). This variability was evident in our samples, with COANCESTRY estimates spanning a broad range across estimators and datasets, and a trend of lower values emerging as SNP count increased. While such variation has been reported in previous studies (Hill and Weir 2011, 2012; Speed and Balding 2015; Galla et al. 2020), 2 specific parent-offspring pairs in our dataset consistently produced lower relatedness values, closer to those expected of second-degree relatives (∼0.25), and were frequently undetected as first-degree pairs in PLINK-KING, Sequoia, and COLONY analyses. These 2 pairs were identified with the medium-density array using COLONY, as potentially COLONY performs best when given a medium-density of genetic markers. While there was no clear biological rationale for this discrepancy, the possibility of sample mislabeling, swapping, or other errors, whether during field collection, DNA extraction, or downstream processing, cannot be entirely ruled out.

Studies have long debated the appropriate usage of relatedness estimators (Hauser et al. 2022). Our comparison of various methods for estimating genetic relatedness revealed several key differences in their strengths and limitations. PLINK-KING provides computationally quick relatedness analyses even when dealing with large datasets (Manichaikul et al. 2010). However, the absence of built-in life-history data integration may reduce its resolution in differentiating specific relationships among closely related individuals in temporal datasets. Our findings also highlighted that missing or ambiguous data posed significant challenges, a concern raised in previous research (Kling and Tillmar 2019; Tillmar and Kling 2025). Furthermore, studies that rely on KING to identify genetically related pairs to filter out of their data, but fail to apply filtering thresholds beforehand, could be problematic (Munshi-South et al. 2016; Theodorou et al. 2018). This limitation may also extend to other relatedness estimators not assessed in this study (de Jong et al. 2024). Similar to PLINK-KING, COANCESTRY lacks the ability to incorporate life-history data, although it offers a broad range of relatedness estimators. Although we focused specifically on comparing trends across arrays, other studies have explored these estimators in greater depth (Attard et al. 2018; Hauser et al. 2022). However, some researchers argue that the foundational equations used in these estimators may not fully capture the complexity of true relationships, potentially limiting their value (Speed and Balding 2015). Moreover, in the absence of life-history data integration, COANCESTRY may not be the best option for temporal studies.

Studies with access to known genetic pairings, age data, or birth and death records can leverage this information to improve accuracy using pedigree-informed analyses. COLONY proved to be effective at identifying our known genetic pairs for all 3 of our datasets. By incorporating a single known maternal pair, COLONY improves its ability to identify parent-offspring and sibling relationships, making it a valuable tool when such prior information is accessible. However, one of COLONY's notable drawbacks is its high computational demand, which has been a concern in previous studies (Hauser et al. 2011). Additionally, COLONY is limited in the types of relationships it can infer, focusing primarily on parentage and sibling relationships (Jones and Wang 2010). COLONY is often compared to Sequoia due to their similarities, and relatively similar accuracy (Huisman 2017; Rentof et al. 2024). Sequoia is generally recognized for being computationally faster, considering a broader range of relationships, but is more conservative in its estimates than COLONY (Huisman 2017; Rentof et al. 2024). Sequoia incorporates biological data such as age, sex, and lifespan, particularly through its “agepriors” argument, which allows for biologically plausible relatedness assignments (Huisman 2017). This integration of life-history information enhances Sequoia's accuracy in detecting genetically related pairs, even across datasets with varying densities. Sequoia additionally includes both the “*Par”* module, which assesses parentage, and the “*Ped”* module, which is a pedigree assessment (Huisman 2017). In our medium-density and high-density datasets, Sequoia's “*Ped”* module failed to identify any full-sibling pairs. Based on discussions with the Sequoia developer and our COANCESTRY results showing high relatedness among our full-sibling pairs, it is possible that Sequoia struggled to distinguish them from duplicates due to their high genetic similarity and a large number of shared SNPs in the denser datasets. Although the *GetMaybeRel* function in Sequoia does not explicitly flag duplicate individuals, we conducted a separate duplicate-checking analysis, which also failed to detect many full-sibling pairs, particularly in the medium-density and high-density arrays. This limitation may stem from Sequoia being optimized for lower-density datasets, where it performs best. However, we were able to identify these pairs using the “*Par”* module without incorporating life history data. In our analysis, Sequoia performed best on the 600-loci dataset that minimized missing data, which was recommended based on Sequoia being trained with datasets consisting of 400 to 600 SNPs (Huisman 2017). This supports the conclusion by Speed and Balding (2015) that the strengths and limitations of the software should be accounted for, and the software should be chosen for the specific dataset at hand. Either the “*Par”* or “*Ped”* module can be used reliably to address research questions when paired with reduced missing data and a relatively low-density of SNPs. Given Sequoia's integration of biological data and its computational efficiency, it appears to be the most reliable method overall in our data, especially considering the computational time limitations of COLONY (Jones and Wang 2010; Huisman 2017; Rentof et al. 2024).

## Conclusion

This study highlights the effectiveness of commercially available medium-density and high-density SNP arrays for assessing genetic relationships in white-tailed deer. The low-density, 600-loci dataset we created provided the most consistent relatedness estimates, while the high-density array offered improved resolution for population structure analysis. The role of filtering thresholds was crucial in optimizing SNP retention, particularly for the high-density array. Sequoia proved to be a highly efficient relatedness estimator when using a low density of SNPs with no missing data. This study showed the importance of integrating life-history data with genomic analyses to enhance kinship accuracy. Properly filtering missing data and selecting the optimal number of loci for a given estimator can further improve precision. Implementation of these recommendations could especially benefit studies with temporal datasets. Low-density SNP arrays with minimal missing data present a promising opportunity for interpreting accurate genetic relationships in wildlife populations. Overall, commercially available medium-density arrays offer a reliable and cost-effective solution for relatedness analysis, especially when paired with proper filtering for tools like Sequoia. Future research investigating the commercial availability of low-density SNP arrays in white-tailed deer and other wildlife species could enhance genetic assessments and optimize management strategies for wildlife populations.

## Supplementary Material

jkag007_Supplementary_Data

## Data Availability

VCFs of the medium-density and high-density array, along with all input files are available on Dryad (https://doi.org/10.5061/dryad.m63xsj4fm). Accompanying scripts and documentation available on GitHub (https://github.com/AlecJChristensen/kinship-detection-snp-density-2025.git). Supplementary Table 1 provides breakdown of minimum and maximum birth years for Sequoia by age class. Supplementary Table 2 provides averages and ranges of COANCESTRY estimates. Supplementary Fig. 2 shows comparison of the “*Par*” and “*Ped*” modules in Sequoia. Supplementary Fig. 3 shows cross-validation error scores from Admixture. Supplementary Fig. 4 shows Admixture analysis plots. Supplementary material available at G3 online.
