# Supplementary material for: Assessing kinship detection: single nucleotide polymorphism array density and estimator comparison in white-tailed deer
Source: G3 (Bethesda). 2026 Jan 25;16(4):jkag007. doi: 10.1093/g3journal/jkag007 (PMC13042285; doi:10.1093/g3journal/jkag007)
Supplement: jkag007_Supplementary_Data [file jkag007_supplementary_data.zip › Supplementary_Material_G3-2025-406343.docx]

**Supplementary Tables and Figure Legends**

**Table S1.** Individuals with unknown ages were assigned minimum and maximum birth years based on their age class. Below is the breakdown of these birth year ranges for each age class.

| Age Class | Sequoia Birth Year Minimum | | | Sequoia Birth Year Maximum |
| --- | --- | --- | --- | --- |
| Adult | | 15 years prior of death year | 2 years prior of death year | |
| Yearling | | 2 years prior of death year | 1 year prior of death year | |
| Fawn | | 1 year prior of death year | 1 year prior of death year | |
| Fetus | | Same as death year | Same as death year | |
|  |  |  |  |  |

**Table S2.** COANCESTRY averages and ranges across seven estimators for parent-offspring (n = 26; excluding problematic pairs), full-sibling (n = 15), problematic parent-offspring (n = 2), and unknown relationship pairs.

| Data Set: | Parent-Offspring | Full-Sibling | Problematic Parent-Offspring | Unknown Individuals | Estimator |
| --- | --- | --- | --- | --- | --- |
| 600-Loci | 0.49 (0.40 - 0.56) | 0.50 (0.47 - 0.53) | 0.26 (0.25 - 0.28) | 0.02 (0 - 0.53) | DyadML |
| 600-Loci | 0.50 (0.42 - 0.56) | 0.50 (0.48 - 0.54) | 0.30 (0.29 - 0.31) | 0.01 (-0.24 - 0.54) | LynchLi |
| 600-Loci | 0.47 (0.37 - 0.55) | 0.47 (0.40 - 0.59) | 0.24 (0.21 - 0.27) | -0.02 (-0.16 - 0.54) | LynchRD |
| 600-Loci | 0.48 (0.40 - 0.56) | 0.50 (0.48 - 0.56) | 0.28 (0.27 - 0.30) | -0.02 (-0.57 - 0.53) | QuellerGt |
| 600-Loci | 0.44 (0.32 - 0.52) | 0.45 (0.36 - 0.60) | 0.21 (0.18 - 0.25) | -0.02 (-0.16 - 0.52) | Ritland |
| 600-Loci | 0.48 (0.39 - 0.55) | 0.50 (0.47 - 0.53) | 0.26 (0.24 - 0.28) | 0.02 (0 - 0.53) | TrioML |
| 600-Loci | 0.50 (0.42 - 0.56) | 0.51 (0.48 - 0.55) | 0.31 (0.30 - 0.31) | 0.01 (-0.29 - 0.54) | Wang |
| Medium-Density | 0.44 (0.35 - 0.50) | 0.47 (0.45 - 0.48) | 0.20 (0.20 - 0.21) | 0.01 (0 - 0.51) | DyadML |
| Medium-Density | 0.45 (0.37 - 0.50) | 0.48 (0.46 - 0.50) | 0.23 (0.22 - 0.23) | -0.01 (-0.32 - 0.52) | LynchLi |
| Medium-Density | 0.42 (0.31 - 0.48) | 0.47 (0.43 - 0.48) | 0.19 (0.19 - 0.20) | -0.02 (-0.08 - 0.51) | LynchRD |
| Medium-Density | 0.42 (0.27 - 0.50) | 0.49 (0.45 - 0.51) | 0.20 (0.19 - 0.21) | -0.02 (-0.44 - 0.53) | QuellerGt |
| Medium-Density | 0.40 (0.30 - 0.47) | 0.45 (0.41 - 0.47) | 0.19 (0.18 - 0.20) | -0.02 (-0.1 - 0.56) | Ritland |
| Medium-Density | 0.44 (0.35 - 0.50) | 0.47 (0.45 - 0.48) | 0.20 (0.2 - 0.21) | 0.01 (0 - 0.51) | TrioML |
| Medium-Density | 0.45 (0.37 - 0.51) | 0.49 (0.46 - 0.50) | 0.23 (0.22 - 0.23) | 0.00 (-0.35 - 0.53) | Wang |
| High-Density | 0.39 (0.22 - 0.47) | 0.45 (0.42 - 0.47) | 0.18 (0.15 - 0.20) | 0.01 (0 - 0.51) | DyadML |
| High-Density | 0.40 (0.23 - 0.48) | 0.46 (0.43 - 0.48) | 0.21 (0.19 - 0.24) | -0.01 (-0.40 - 0.52) | LynchLi |
| High-Density | 0.37 (0.21 - 0.44) | 0.44 (0.40 - 0.47) | 0.17 (0.15 - 0.19) | -0.02 (-0.10 - 0.50) | LynchRD |
| High-Density | 0.36 (0.14 - 0.46) | 0.46 (0.42 - 0.50) | 0.14 (0.10 - 0.18) | -0.02 (-0.40 - 0.54) | QuellerGt |
| High-Density | 0.35 (0.22 - 0.42) | 0.42 (0.36 - 0.46) | 0.16 (0.15 - 0.18) | -0.02 (-0.13 - 0.74) | Ritland |
| High-Density | 0.39 (0.22 - 0.47) | 0.45 (0.42 - 0.47) | 0.18 (0.15 - 0.20) | 0.01 (0 - 0.51) | TrioML |
| High-Density | 0.40 (0.22 - 0.48) | 0.47 (0.43 - 0.49) | 0.21 (0.18 - 0.24) | -0.01 (-0.43 - 0.52) | Wang |

**Figure S1.** Sampled white-tailed deer with known relatedness, including parent-offspring and full-sibling pairs, as well as individuals with unknown relatedness in Southeast Minnesota.

**Figure S2.** Comparison of the “*Par*” and “*Ped*” modules in Sequoia in identifying known genetically related parent-offspring pairs (n = 28) and full-sibling pairs (n = 15) across our three datasets (600-Loci, Medium-Density, High-Density). “*Par*” is a parental assessment and “*Ped*” is a pedigree assessment.

**Figure S3.** Admixture cross-validation error scores for 600-loci, medium-density, and high-density data sets across ancestral population clusters (K = 1 to K = 10).

**Figure S4.** Admixture (K = 2) analysis results for white-tailed deer genotyped across three datasets: (a) 600-loci dataset, (b) medium-density array, and (c) high-density array. Cross-validation error supported K = 2 for the high-density array but supported K = 1 for the medium-density array and the 600-Loci data set.
