# Supplementary figures and images for: Assessing kinship detection: single nucleotide polymorphism array density and estimator comparison in white-tailed deer
Source: G3 (Bethesda). 2026 Jan 25;16(4):jkag007. doi: 10.1093/g3journal/jkag007 (PMC13042285; doi:10.1093/g3journal/jkag007)

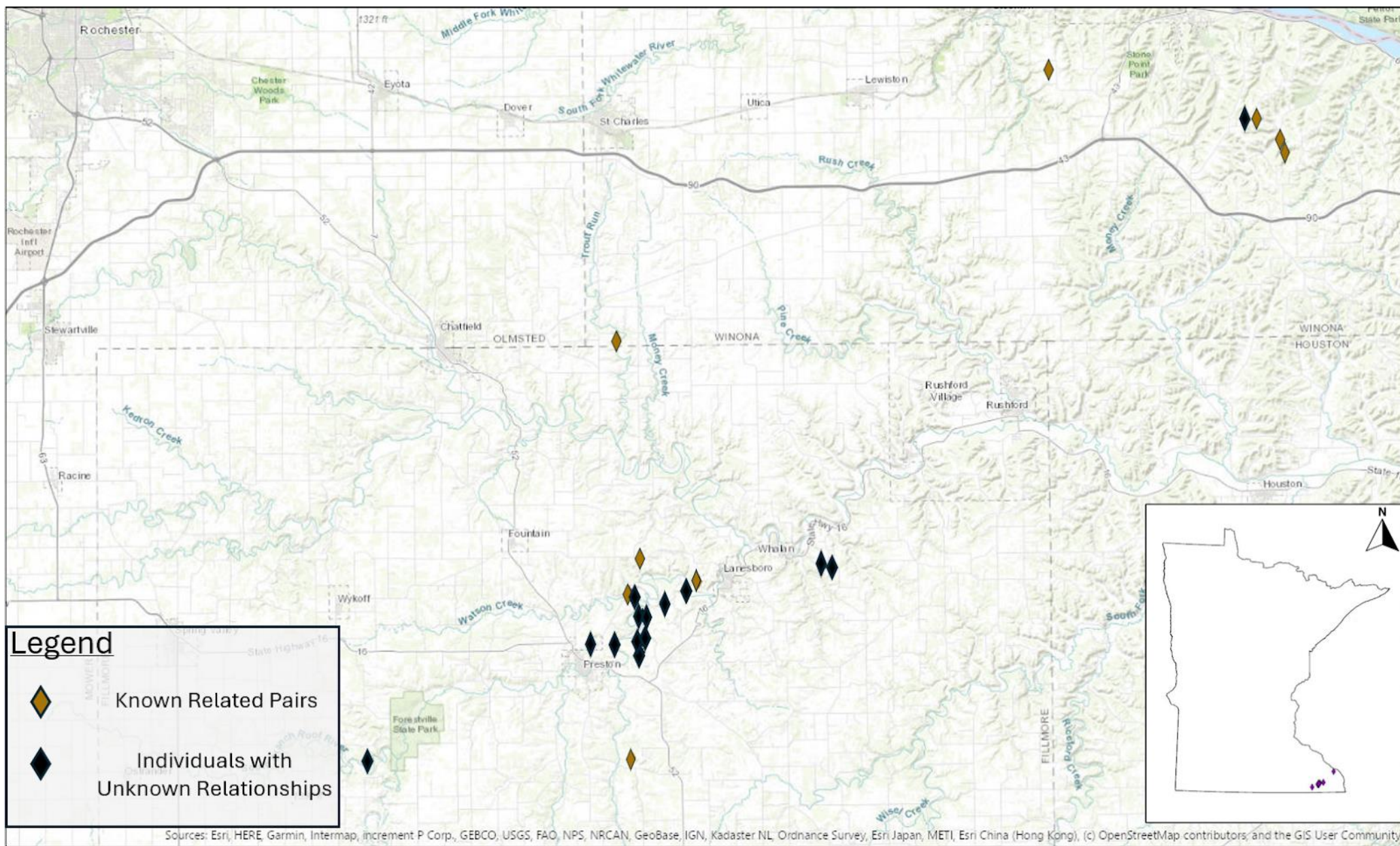

Supplement: jkag007_Supplementary_Data [file jkag007_supplementary_data.zip › Figure_S1_G3-2025-406343.pdf]

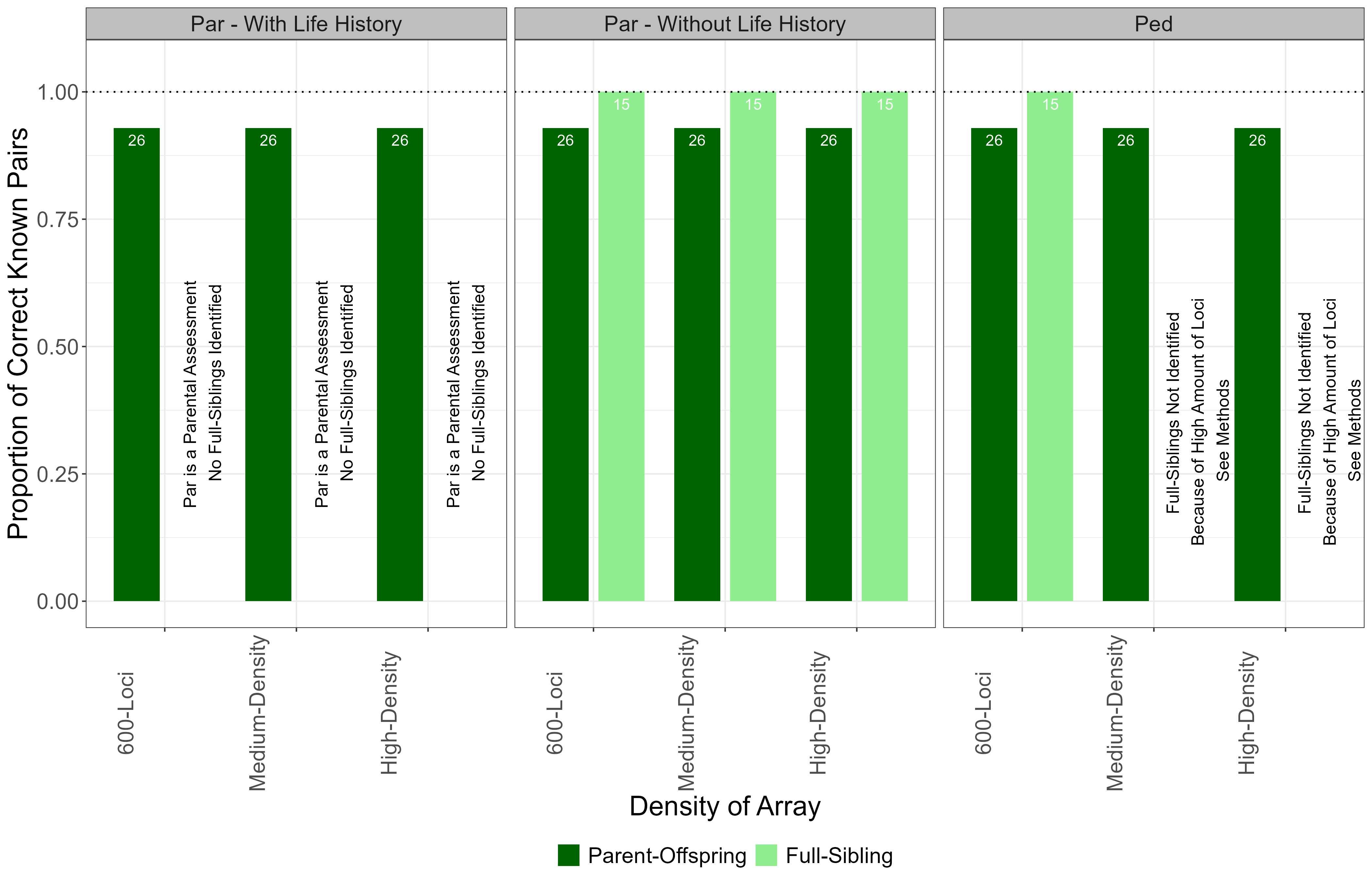

Supplement: jkag007_Supplementary_Data [file jkag007_supplementary_data.zip › Figure_S2_G3-2025-406343.tif]

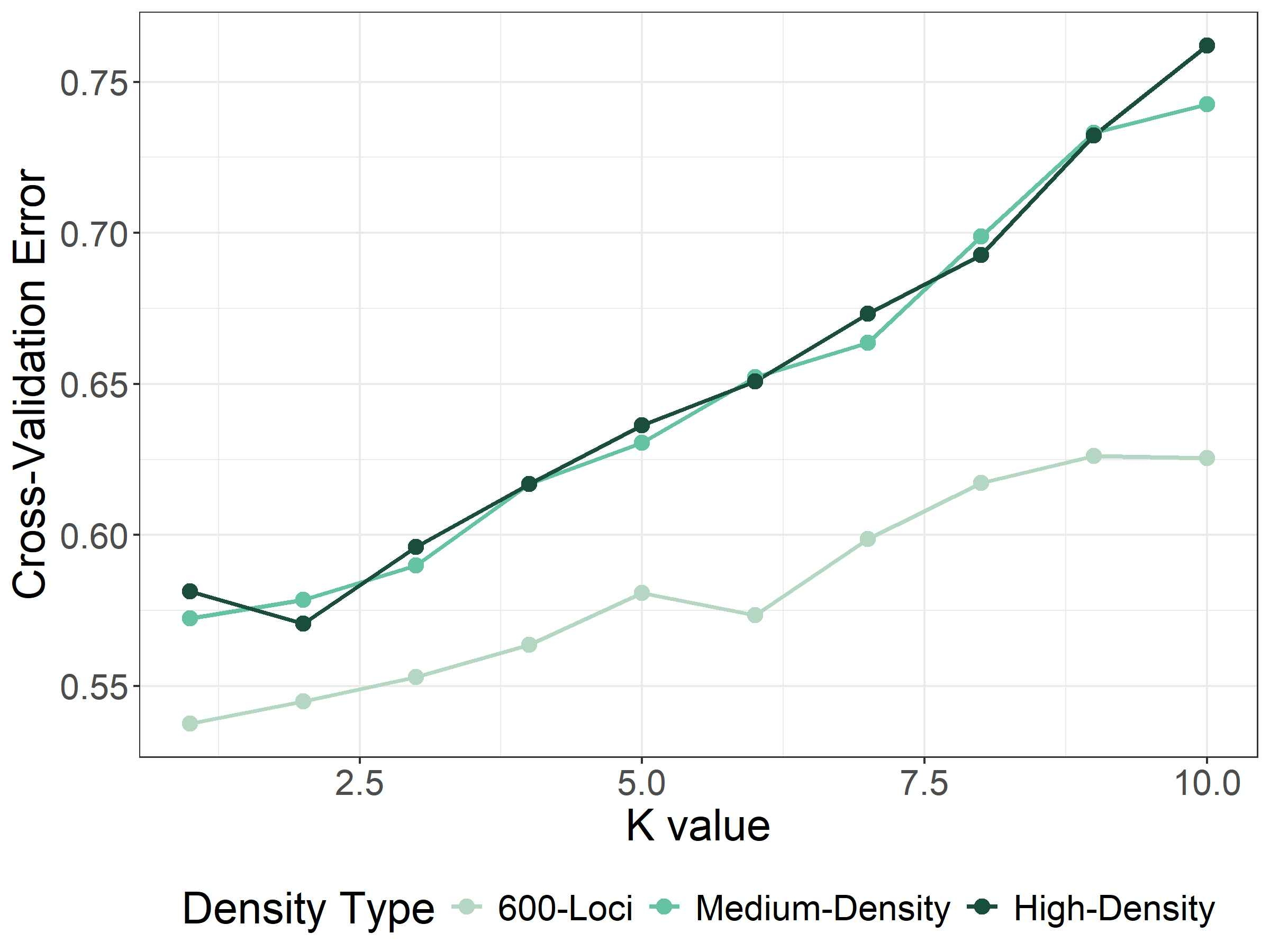

Supplement: jkag007_Supplementary_Data [file jkag007_supplementary_data.zip › Figure_S3_G3-2025-406343.tif]

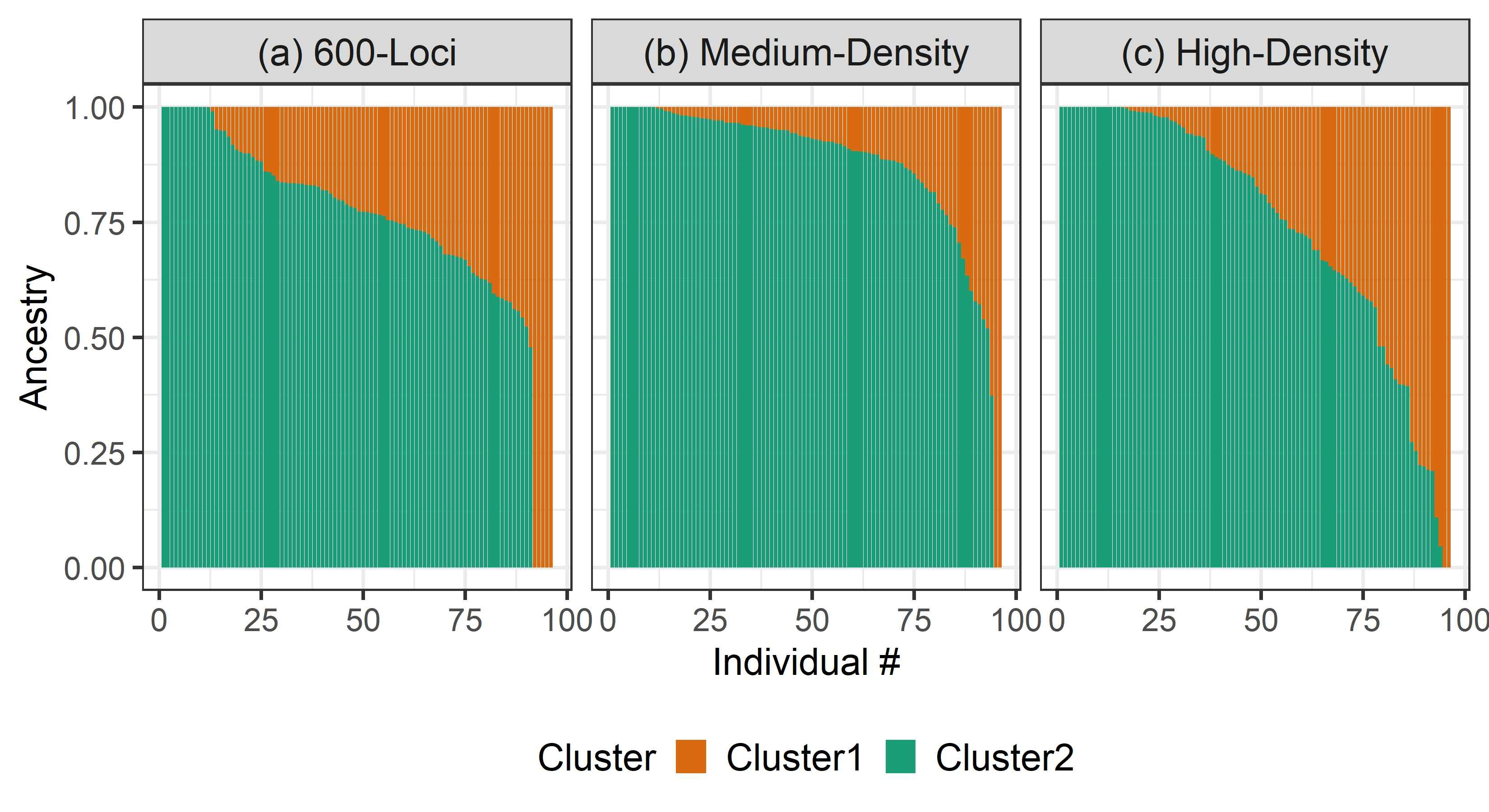

Supplement: jkag007_Supplementary_Data [file jkag007_supplementary_data.zip › Figure_S4_G3-2025-406343.tif]
